# The importance of regeneration processes on forest biodiversity in old-growth forests in the Pacific Northwest

**DOI:** 10.1098/rstb.2023.0016

**Published:** 2024-05-27

**Authors:** Hoang Luu, Janneke Hille Ris Lambers, James A. Lutz, Margaret Metz, Rebecca S. Snell

**Affiliations:** ^1^ Environmental and Plant Biology, Ohio University, Athens, OH 45701-2978, USA; ^2^ ETH Zürich, Zurich 8006, Switzerland; ^3^ Wildland Resources, Utah State University, Logan, UT 84322, USA; ^4^ Lewis & Clark College, Portland, OR 97219, USA

**Keywords:** seed production, forest composition, forest gap model, old-growth forest

## Abstract

Forest diversity is the outcome of multiple species-specific processes and tolerances, from regeneration, growth, competition and mortality of trees. Predicting diversity thus requires a comprehensive understanding of those processes. Regeneration processes have traditionally been overlooked, due to high stochasticity and assumptions that recruitment is not limiting for forests. Thus, we investigated the importance of seed production and seedling survival on forest diversity in the Pacific Northwest (PNW) using a forest gap model (ForClim). Equations for regeneration processes were fit to empirical data and added into the model, followed by simulations where regeneration processes and parameter values varied. Adding regeneration processes into ForClim improved the simulation of species composition, compared to Forest Inventory Analysis data. We also found that seed production was not as important as seedling survival, and the time it took for seedlings to grow into saplings was a critical recruitment parameter for accurately capturing tree species diversity in PNW forest stands. However, our simulations considered historical climate only. Due to the sensitivity of seed production and seedling survival to weather, future climate change may alter seed production or seedling survival and future climate change simulations should include these regeneration processes to predict future forest dynamics in the PNW.

This article is part of the theme issue ‘Ecological novelty and planetary stewardship: biodiversity dynamics in a transforming biosphere’.

## 1. Introduction

Forests with high species diversity have been shown to have higher ecosystem functioning and higher levels of ecosystem service provisioning [1–3]. For example, high tree species diversity maximizes resource use, leading to higher growth and productivity [4]. Forests with phylogenetically distinct species have also been found to have increased resistance to herbivory damage [5] and high tree diversity can also increase resilience to disturbances and climate change [6]. However, climate change is expected to alter tree species composition (e.g. [7,8]), via changes to mortality [9–11], growth [12,13], competition [14] and recruitment [15]. Thus, it is critical to identify how environmental factors influence each of these processes at the individual species level, so that we may understand the drivers of current species composition, and better predict how species composition may change in the future.

Regeneration and recruitment are critical processes for determining species composition. Here we define regeneration as the processes that add new seedlings to a population (i.e. flowering, seed production, germination and seedling survival) while recruitment is the process by which seedlings grow to exceed a minimum size diameter at breast height (DBH) threshold to become a sapling [16]. Each of these stages is influenced by a variety of biotic and abiotic factors, making it challenging to determine how altering one factor would influence species composition. For example, seed production is highly sensitive to individual tree size and weather [13,17]. In North American trees, annual seed production was directly influenced by temperature and moisture deficit, but also indirectly, via climate-induced changes in growth [13].

Evidence of climate change impacts on seed production is already being observed. For example, cone production in *Pinus edulis* has declined by 40% over the past decade due to increasing temperatures [18]. This reduced seed availability has resulted in lower tree recruitment of *P. edulis* following drought- and insect-induced mortality of the overstorey. Changes in temperature and precipitation will also influence germination rates and seedling survival, both of which directly impact plant recruitment [19]. For instance, germination of *Pinus ponderosa* increased with warmer temperatures [20], although another study found a decrease in recruitment for the same species [21,22]. This variability results in a diverse regeneration response under the influence of future climate change. In another example, *Tsuga mertensiana* has low seedling survival during droughts due to their shallow roots [23], and may have a negative regeneration response under future climate conditions. Altogether, each step in the regeneration process can contribute to a phenomenon known as *recruitment limitation*. Especially under climate change, it is uncertain if recruitment limitation will become more important in forests, and if this will be due to changes in seed production and/or seedling establishment [13].

Numerous theoretical models highlight the critical role of regeneration processes in shaping forest ecosystems. Competition–colonization models propose that the coexistence of tree species is influenced by their competitive abilities for resources and colonization capacities in different environmental niches [24]. Species with strong competitive traits may dominate resource-rich areas, while others with efficient colonization mechanisms thrive in less favourable conditions. Storage effect models, tailored to tree dynamics, underscore the importance of seed and seedling banks and the temporal variability of environmental factors [25]. These models suggest that diversity in regeneration strategies among species, such as variation in seed production, germination requirements or seedling competition, contribute to the coexistence of tree species by capitalizing on changing conditions over time. It is therefore likely that the relative importance of each of these processes varies by species, and over time.

One of the challenges with understanding the relative importance of regeneration is that it takes years for changes in a forest overstorey to become apparent following changes to regeneration. Moreover, many other processes can influence the species composition of a forest, such as growth, competition and mortality. Dynamic vegetation models (DVMs) are one approach to overcome this limitation, allowing us to quantify the relative importance of different processes on forest biodiversity and forest structure [26]. DVMs simulate the many known abiotic and biotic processes in a forest (resource availability, individual tree growth, tree mortality, recruitment, etc.), and can do so over long time scales and different climatic conditions [27]. DVMs include a broad group of models, including dynamic global vegetation models (DGVMs), forest landscape models and forest gap models [26]. Forest gap models simulate competition for light and other resources at the scale of a forest stand [28]. Gap models require climate, soil and species-specific performance traits as input. Some of the performance traits include growth rates as a function of light, temperature and moisture, shade tolerance and maximum height. Using such models can help us gain a deeper understanding of how changes in one of the processes can impact the biodiversity of a forest.

However, all models make assumptions about specific processes due to insufficient data, a lack of theoretical understanding, or computational limitations. One of the common assumptions in forest gap models relates to the regeneration and recruitment processes. In forest gap models, the process is referred to as ‘sapling rain’ and assumes that seeds for all species are always available and new saplings are added into the model if certain environmental conditions are met [29]. A recent review of forest models found that the simulation of regeneration processes is limited by structural model constraints, species-specific parameterizations (e.g. seed production) and knowledge gaps [25]. Establishment parameters were also identified as critically important for the simulation of tree basal area even under the current climate [30]. Improving the representation and parameterization of regeneration will likely become even more critical to accurately capture regeneration patterns over time and in response to climate change [31].

Here, we improve the representation of the regeneration process in a forest gap model by adding seed production and seedling survival, as functions of weather and biotic conditions. We focus on old-growth forests in the Pacific Northwest (PNW) where long-lived tree species can be found and long-term data on seed production (i.e. since the 1960s) is available [13]. We use the forest gap model ForClim because it has been successfully parameterized for forests in the PNW [32]. By comparing the different model versions, with different regeneration processes included or excluded, we answer the following research questions:

1. What is the relative importance of the regeneration process (seed production and seedling survival) for forest diversity?
2. How do the regeneration processes influence species-level basal area?

## 2. Methods

ForClim is a cohort-based forest-gap model developed to simulate tree establishment, growth and mortality as a function of environmental and stand-level properties at annual resolutions [33–35]. One of the assumptions in ForClim is that new saplings at 2.54 cm DBH are always available and will establish if species-specific environmental conditions are met (e.g. soil moisture, winter temperature, growing degree days, available light) [34]. This approach has been referred to as the ‘sapling rain’ method [29], and is a valid assumption if seed production or availability of seedlings do not limit recruitment [29]. However, the data suggests that most plant species are seed-limited, which can limit recruitment [36,37]. ForClim v4.0. is the base version used in this study [38], and see electronic supplementary material §1 for more details about ForClim.

### (a) New seed production module

We modified ForClim to include the additional processes of (i) annual seed production and (ii) annual seedling survival until reaching the sapling stage. The base function for seed production is a product of exponential functions because seed production is known to be exponentially related to tree size [13,17,39]. An individual tree produces seeds based on the following equation:

2.1 $$Si=\;e^{\left(a*\mathrm{DBH}\;+\;b*{\mathrm{DBH}}^{2}\;+\;c*\mathrm{weather}1\;+\;d*\mathrm{weather}1^{2}\;+\;e*\mathrm{weather}2\;\ldots .\right)}\;,$$

where *Si* is the number of seeds produced by an individual tree, DBH is the diameter at breast height of the tree, ‘weather#’ are various weather variables, and each coefficient (*a*, *b*, *c*, etc.) is a species-specific value (electronic supplementary material §2). Weather variables that are known to influence seed production were incorporated into the equation. This includes spring and summer temperatures from the current and past two years, along with the water deficit observed in the current year [13,39]. Annual seed production and tree size data came from the MASTIF network [13] and Wind River Forest Dynamic Plot [40]. Weather data of the sites where the plots are located comes from the Parameter-elevation Regressions on Independent Slopes Model (PRISM) [41]. Of the 18 species and two sub-species that were already parameterized in ForClim, 12 of the species had seed data to parameterize seed production equations (electronic supplementary material, table S3, figure S1). The six species that were excluded in the updated seed model are rare in the PNW and were also not observed in the Forest Inventory Analysis (FIA) data. We employed a backwards selection method to parameterize the seed production equation. This approach starts with a fully saturated model with all relevant predictor variables and iteratively eliminates the least significant variable until a model with only significant predictors is achieved.

After calculating annual seed production per tree (equation (2.1)), total seed production (St) is calculated for the patch for each species (i.e. seed production is calculated for each cohort based on the DBH, then multiplied by the number of individuals in the cohort (*n*_cohort_), and then summed up among cohorts of the same species). In addition to the variable seed production from trees within the patch, a constant number of seeds (seed_const_ = 100 by default) is added to the patch each year from all species. This parameter is meant to represent seeds that have dispersed into the patch via long-distance seed dispersal. ForClim does not have patch-to-patch interactions and the seed_const_ parameter is needed as a way for a new species to be introduced into the patch.

2.2 $$St={\mathrm{seed}}_{\mathrm{const}}+\sum_{n}^{\mathrm{Cohorts}}\left(\mathrm{Calculated}\;\mathrm{Seeds}\;\mathrm{Produce}d_{n}*n_{\mathrm{cohort}}\right).$$

Next, the number of seedlings is calculated from the total number of seeds produced. Although available light, nutrients, temperature and other limiting factors can influence germination rates [36], a lack of data limited our ability to create a species-specific germination equation. Thus, the proportion of seeds that germinate into seedlings was calculated as a constant (*gr* = 0.01) of seeds produced [29].

2.3  Seedlings =(St)∗gr.

The number of seedlings that survive is based on their species-specific responses to environmental conditions, plus some stochasticity. We chose to model seedling survival using the same data (i.e. FIA seedlings) and same equations as Canham and Murphy [42]. Annual seedling survival probability was calculated with a negative exponential function (electronic supplementary material §3)

2.4 $$\mathrm{Seedling}\;\mathrm{survival}\;=\;\exp\left[(-c_{a}{\mathrm{BA}}_{t}^{c_{b}})-\frac{1}{2}{\left(\frac{\mathrm{MAT}-t_{a}}{t_{b}}\right)}^{2}-\frac{1}{2}{\left(\frac{\mathrm{WD}-w_{a}}{w_{b}}\right)}^{2}\right],$$

where BA*_t_* is the total basal area in the patch, MAT is the mean annual temperature, and WD is water deficit (mm). The survival equation has species-specific survival parameters for basal area (*c_a_*, *c_b_*), mean annual temperature (*t_a_*, *t_b_*) and water deficit (*w_a_* and *w_b_*).

The number of seedlings that survive for that year is determined by a randomly generated value pulled from a beta distribution, where the calculated seedling survival (equation (2.5)) is the mean and with a selected beta distribution variance (Beta_var_ = 0.1). Although the time it takes for a seedling to recruit into the sapling stage may depend on available resources [43], we set the seedling survival time (*Ts*) to 10 years for all species due to data limitations. A seedling is considered a sapling when it reaches a DBH greater than 2.54 cm. Ten years was also the assumption used for creating the seedling survival equations (i.e. the average time it takes for seedlings to become a sapling) and corresponded to the average length of time between FIA resampling dates.

2.5 $$\#\;\mathrm{of}\;\mathrm{Seedling}s_{Ts+1}=\;{\left(\#\;\mathrm{of}\;\mathrm{Seedlings}\right)}_{Ts}\;*\;{\left(\mathrm{Beta}\;\mathrm{Distibution}(\mathrm{Seedling}\;\mathrm{Survival}\;\text{\%},\;\;{\mathrm{Beta}}_{\mathrm{Var}})\right)}_{Ts}\;.$$

Finally, a new sapling cohort is added to the patch if there is available space in the simulated 800 m^2^ forest patch. Available space is determined by using the original ForClim model's establishment routine where the number of new saplings that can be added is determined by the number of existing trees in the model and environmental conditions such as available light at the forest floor [44]. Browsing is an integral component of the ForClim model, influencing seedling establishment through species-specific browsing sensitivity. Given the limited data on browsing at the site, the default value for browsing pressure (kBrPr = 0.2) was uniformly applied across all simulations [38]. Saplings are randomly selected from the pool of available saplings until the number of new saplings determined by ForClim is fulfilled. Saplings that are not added to the model are removed. We opted against calibrating the model using the sensitivity analysis, in line with ForClim's philosophy. Our focus is on understanding the main drivers behind individual processes, rather than aligning the results perfectly with observed patterns.

### (b) Model application

We chose to simulate old-growth forests in the PNW that have no record of disturbance or management. From the FIA data, we selected plots in Oregon and Washington state, that had an estimated stand age of more than 500 years by FIA records. This yielded 2106 unique 700 m^2^ plots. We then randomly selected 10% of those FIA plots to simulate (*n* = 210 sites). The weather and soil conditions at those FIA plots are provided as model input. Since the simulations start from ‘bare ground’, we need to add seeds at the very start of the simulation. Thus, 100 000 seeds of every species are added to each patch for a set number of years (i.e. seed initialization time, default set at 10 years). Simulations were run for 500 years to represent the stable state of the forest [33] and to match the FIA stand records. Historical weather data came from PRISM (1895–2021) and was randomly resampled with replacement, to create the 500-year weather time series. Soil water-holding capacity for each stand was estimated with the Global Assessment of Water Holding Capacity of Soils [45]. Model evaluation was done by (i) comparing steady-state simulations with old growth FIA data and (ii) a sensitivity analysis with the new seed production processes and parameters added into the model.

### (c) Model evaluation

Each of the 210 simulated stands was simulated with both the original ForClim model and the updated ForClim model, which included seedling survival and seed production as described above. We compared the simulated basal area per hectare for each species at the simulated year 500 between the two model versions, as well as with the FIA data. Basal area per hectare was selected as a metric of comparison because it is the most available measurement provided by the observed FIA data. Here species abundance is basal area density of a species and species composition is the relative abundance of all the species in a stand. The Euclidean distance, computed through the ‘vegdist’ function from the ‘vegan’ package in R [46], serves as a metric for comparing species composition between the FIA data and simulated results. This comparison also addresses our research questions, as we can examine how the inclusion of regeneration processes impacts the simulation of individual species and species composition.

### (d) Sensitivity analysis

The sensitivity analysis here aimed to comprehensively assess both the understanding of the new seed production functionality and the model's sensitivity to variations in the new parameter values. A structural sensitivity analysis with the updated model was done where parts of the seed production processes were included or removed. The goal was to identify the relative importance of different seed production components on the regeneration processes for forest biodiversity. The model versions included: (i) the complete updated model with seed production as a function of DBH and weather, (ii) the updated model with seed production as a function of DBH only, (iii) the updated model with seed production as a function of weather only, and (iv) the updated model with seed production set as a constant (100 seeds for each individual every year). The 210 stands were run with the four model variants giving us a total of 840 simulations. The basal area of each species in the stand at year 500 was recorded and used to calculate the Simpson diversity index of the stand for model evaluation.

Second, we did a sensitivity analysis with the full updated model to quantify the uncertainty and relative importance of the newly added parameters on the output of the model (protocol as described in Snell [47]). The weather parameters were excluded from this analysis because they were derived from existing data. The parameters include seed initialization time (*T*_int_), seed constant (seed_const_), germination rate (*gr*), sapling growth time (*T*_s_) and survival beta variance (Beta_var_). *T*_int_ is the duration for which seeds are introduced into the simulation model at the start, seed_const_ is the constant number of seeds introduced into the patch each year, *gr* is the germination probability that a seed becomes a seedling, *T*_s_ is the time it takes for a seedling to grow into a sapling, and Beta_var_ is the additional variability in seedling survival rates. Latin hypercube sampling was used to create 100 random parameter sets that covered the entire parameter space. The seed initialization time ranged from 1 to 20 years, the seed constant ranged from 10–1000 seeds per year, the seed germination rate ranges from 1 to 10% [29], the seedling to sapling growth years range from 5 to 15 years [48,49], and the survival beta variance ranged from 0.02 to 0.2. The correlation coefficient of the basal area of each species with each of the selected parameters was calculated. The mean square values of each parameter obtained through the analysis of variance for the Simpson diversity index offer insights into the proportion of variability attributed to each parameter. All analyses comparing the basal area and Simpson diversity index of the model with the FIA data were performed using R Statistical Software version (v4.2.2; R Core Team [50]).

## 3. Results

**Figure 1.**
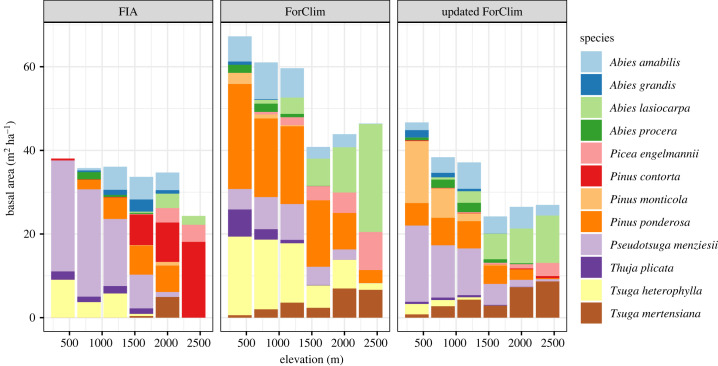
Total basal area along an elevation gradient for each species from FIA data, the original ForClim model, and the updated ForClim model. The minimum DBH from the FIA is 12.7 cm and the model is 1.27 cm as the minimum. The updated model included seed production and seedling survival, where seed production was a function of DBH and weather. Each bar is the average total basal area of the species in a forest stand divided into 400 m bins.

**Figure 2.**
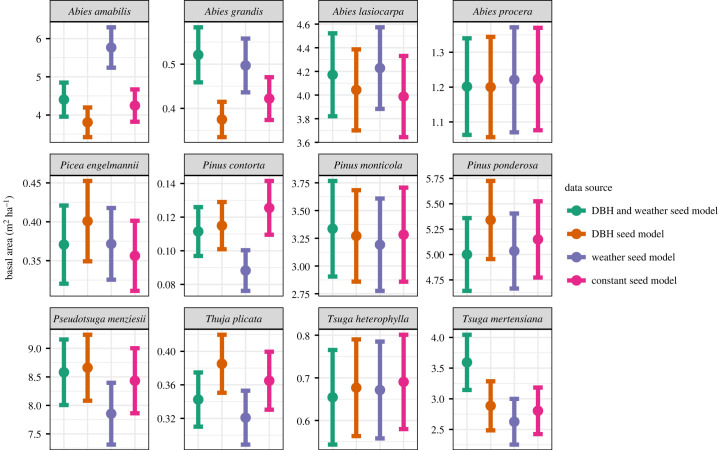
Mean (±s.e.m.) basal area of each species across all plots from different versions of the updated model that varied in how seed production was calculated. The models have seed production as a function of (i) DBH and weather, (ii) DBH, (iii) weather and (iv) a constant. All of the models used the same seedling survival function.

Similar to previous studies in the PNW [32,34], ForClim captured broad patterns in species composition along elevational gradients (figure 1; electronic supplementary material, figure S3). Adding regeneration processes into ForClim decreased the simulated total basal area, which more closely matched the observed data (figure 1). Although differences in simulated species composition were more nuanced, ForClim models with regeneration processes generally provided a better match to the observed FIA species compositions (figure 1; electronic supplementary material, figure S7, table S5). Specifically, compared to the original model and the FIA data, the updated model more accurately simulated the observed dominance of *Pseudotsuga menziesii* (figure 1; electronic supplementary material, figure S3). Other improvements as a result of adding regeneration included the reduced dominance of *Tsuga* species and *Picea engelmannii* at high elevations*.* However, the simulated occurrence of *Pinus ponderosa* and *Thuja plicata* decreased when the models simulated regeneration processes, compared to the original ForClim which is more accurate. There were also a few species that were consistently underrepresented in the simulations (e.g. *Abies grandis*), regardless of whether models simulated regeneration processes or not. All species were insensitive to how seed production was calculated, since the basal area of each species fell within the standard error range for each seed production method (i.e. as a function of weather, DBH or a constant value; figure 2).

While there were species-specific differences when adding regeneration processes, the mean Simpson diversity index did not differ between the ForClim model versions (electronic supplementary material, figure S4), all of which were much higher than what is observed in the FIA stands. Numerous stands in the FIA data emerged as monocultures without any management (indicated by a Simpson diversity index of 0), thereby contributing to a reduced mean FIA Simpson diversity. It is noteworthy that ForClim simulations seldom replicated the occurrence of monocultures.

Of the variables used in the sensitivity analysis of the updated model, sapling growth time had the highest correlations with basal area across all species (some positive and some negative; electronic supplementary material, figure S8) and explained 83.6% of the variation in the Simpson's diversity index (table 1). The seed constant parameter in the model explained 5.7% of the variance in diversity and the other variables tested explained <5% of the variation (table 1).

**Table 1.** Partition of variation of the response variable (the Simpson diversity index) as a function of the newly introduced model parameters, seed initialization time, seed constant, germination rate, sapling growth time and survival beta variance. An analysis of variance was done and the proportion of the mean squares from each of the parameters from the analysis gives the percent of variance explained. More results in electronic supplementary material, table S5.

parameter	% of variance explained
seed initialization time	1.9
seed constant	5.7
germination rate	3.2
sapling growth time	83.6
seedling survival beta variance	3.6

## 4. Discussion

In this study, we found that seedling survival exerted a more pronounced influence on forest diversity than seed production, and observed a significant correlation between sapling growth time and species-level basal area. For tree species in the PNW, the effects and importance of including the regeneration process varied by individual species, with some species increasing in abundance and others decreasing once seed production and seedling survival were included. The diversity predicted by the forest model did not match the FIA data and our results show that adding regeneration processes to the model did not improve it (as quantified by the Simpson diversity index in electronic supplementary material, figure S4). However, the inclusion of regeneration processes did lead to improved predictions of species composition, as evidenced by the reduced Euclidean distance between the FIA data and the updated model (electronic supplementary material, table S6). Below, we will discuss in more detail (i) the importance of recruitment limitation for species composition, (ii) the effects of variation in seed production, and (iii) model and parameter uncertainties.

### (a) Regeneration and recruitment limitation and forest structure

Our results suggest that regeneration processes do limit the abundance of trees in a forest but in a species-specific manner. When using the ‘sapling rain’ method in the original ForClim (i.e. that assumes unlimited seed and seedlings), the simulated forests had a much higher basal area, and individual species were typically found across a wider range of elevations when compared to the FIA data (figure 1; electronic supplementary material, figure S3). Adding seed production and seedling survival limited the abundance of simulated trees and also shifted species composition to more closely match the observed FIA data, suggesting that these regeneration processes play an important role in determining species composition. One of the major improvements was the simulation of *P. menziesii,* where the updated ForClim model simulated a higher basal area at lower elevations that was more similar to the observed data. Compared to other species, seed production (electronic supplementary material, figures S1) and seedling survival (electronic supplementary material, figures S2) of *P. menziesii* is similar to many other species but species dominance varied across elevations. One advantage that *P. menziesii* has is its relatively fast growth rate after it has established as a sapling (electronic supplementary material, table S1). Having a faster growth rate can lead to larger trees and higher seed production earlier than other species, which can limit establishment from their competitors. The combination of high seed production and seedling survival, with the original ForClim growth processes, helped *P. menziesii* to dominate when simulating a stand developing from bare ground. However, the dominance of *P. menziesii* may decline over time as it is less shade tolerant and generally does not recruit well under itself [51]. Given that the FIA forests are estimated to be 500 years old, we stopped our simulations at the same age and extending the simulations beyond that time would predictably lead to a shift in species compositions towards more shade tolerant species (electronic supplementary material, figures S5 and S6).

The other significant improvement in the updated model was the decrease in simulated dominance of other species such as *Tsuga heterophylla*. The decrease in *T. heterophylla* is likely due to a combination of a slower growth rate, a larger minimum size of reproduction (electronic supplementary material, tables S1 and S2), and a lower seedling survival rate when compared to other species (electronic supplementary material, figure S2). *Tsuga heterophylla* does have higher shade tolerance, so the initial recruitment limitation may eventually lead to a steadier recruitment rate [51]. However, there were other species whose ForClim abundances continued to poorly match FIA abundances, even after updating the regeneration processes. This may have been due to other processes that were not included. For instance, the unexpectedly high abundances of *Abies amabilis* in ForClim could be attributed to the absence of simulated disturbances [52].

We also found that seedling survival was more important than seed production for recruitment limitations since changes to how seed production was calculated did not have a significant impact on the simulation outputs (figure 2; electronic supplementary material, figure S4). Previous studies have also found seedling survival to be very sensitive to environmental factors; *Pinus ponderosa* seedling mortality increased by 5.8% per °C above its normal temperature [53]. *Picea engelmannii* and *Abies lasiocarpa* seedlings subject to high light levels and increased water stress had over 80% mortality during their first year of growth [54]. The decrease in basal area with the regeneration update suggests that the ‘sapling rain’ assumption used in many DVMs is likely overestimating the success of species that would be limited by regeneration processes [29]. The sensitivity of seedlings to environmental conditions is also likely to become more important under future climate change and is a process that should be considered in all forest models that aim to simulate climate change impacts.

### (b) The effects of variation in seed production

We were surprised to find that differences in seed production alone did not have a large impact on simulated tree diversity. Specifically, predicted forest diversity was similar when seed production was set at a constant value, or when seed production varied by DBH and/or weather. Theoretically, a species with very high seed production could overcome low seedling survival. The seeds from small-seeded species are more abundant than large-seed species in a forest, but that abundance advantage has been observed to be balanced out by seedling survival processes [55]. However, that abundance advantage was not observed in our models where the final simulated basal area of each species was the same regardless of the seed production processes assumed (figure 2). This choice of seed production method could vary the total number of seeds produced by a species, by orders of magnitude (electronic supplementary material, figure S5). For example, *Tsuga mertensiana* produced approximately 2000 seeds each year in a stand when seed production was set as a constant, but was producing approximately 60 000 seeds when seed production was set as a function of DBH and weather (electronic supplementary material, figure S5). Despite this seed number advantage, the simulated basal area of *T. mertensiana* was the same between simulations (electronic supplementary material, figure S6). Our results suggest that seed availability may not be a limiting factor for old-growth forests in the PNW and that the availability of a suitable environment for germination and seedling survival is more important.

However, seed production is sensitive to climate and seed production equations of all of our species included at least one temperature variable (spring and/or summer temperatures; electronic supplementary material, table S3). Across genera, seed production in North American conifers was found to be driven by an absolute difference in summer temperatures (i.e. the *Δ*T model; [56]) which has led to the hypothesis that the difference in summer temperatures would remain consistent despite climate shifts [57]. In support of this hypothesis, LaMontagne *et al*. [56] found no change in seed production variation over time due to recent climate warming; however, others have found an increase in variation for Pinaceae over recent decades [58]. Based on our results, it is the seed production failures (rather than the seed increases) that are most likely to influence diversity and lead to shifts in species composition.

### (c) Model and parameter uncertainties

A continued limitation of the ForClim model, regardless of whether it included updated regeneration processes, was its ability to accurately simulate diversity. The Simpson diversity index calculated for the base model was higher compared to the index calculated from the FIA data (electronic supplementary material, figure S4). The updated model did not improve diversity estimates, however, nor did it make them worse. This difference is likely due to the assumptions used in the models. Both the original ForClim and the updated model assume that all species can enter the stand every year (i.e. the sapling rain assumption assumes all species can enter as saplings if environmental conditions are suitable, and the simulation of seed production assumes that a small number of seeds from all species disperse in every year). The assumption for seeds was intended to represent long-distance seed dispersal between forest stands and to allow for new species to establish. However, this assumption may have also led to higher diversity since monocultures were rarely simulated. Seed dispersal limitations have been observed to lower the diversity of forests and could be a process added in future models to further filter species establishment [59].

It should also be noted that ForClim was not designed to simulate large disturbances, such as diseases, insects or fire. However, simulating the species composition of a forest after a large-scale disturbance, such as fire, will depend on seed production, seedling survival, and if any of the species have special fire adaptations for reproduction such as serotinous cones. Thus, improving the simulation of seed production and seedling survival is an essential first step, before disturbances are considered. Furthermore, while it is recognized that disturbances, such as fire and drought, can affect seed production and survival in a species-specific manner, our improvements are constrained by insufficient empirical data to appropriately parameterize these relationships.

Introducing the new regeneration processes added parameters and introduced new uncertainties. The sensitivity analysis was able to identify seedling-to-sapling growth time as the most important parameter for simulating tree diversity. Having a longer seedling-to-sapling growth time means that each species spends more time as a seedling, and each year their seedling pool is further reduced by the seedling survival equations. This can have a large negative impact, especially for species with lower seedling survival rates (e.g. *Abies lasiocarpa*). To improve the accuracy of forest simulation modelling, future efforts should focus on quantifying these seedling dynamics. More specifically, the time it takes for a seedling to grow into a sapling can depend on many factors such as the species of tree, seedling quality, environmental conditions and plant competition [48]. Given the importance of the seedling stage for forest diversity, quantifying these seedling-to-sapling relationships for each species as a function of environmental variables will be an important area of future research. The incorporation of regeneration processes in dynamic vegetation models is also an important advancement towards the broader objective of simulating disturbed landscapes within the context of global change. The interaction between climate change, disturbances and forest recovery may depend on regeneration dynamics. For example, increasing fire frequency and intensity are projected for many parts of western and northern parts of North America [60]. A recent study demonstrated that mast seed production in *Picea glauca* is positively correlated with fire the year before [61], as both fire and seed production are synchronized by El Niño–Southern Oscillation events. Continuing work in this area will significantly contribute to improving our understanding and stewardship of biodiversity amid human-driven biosphere transformation.

## 5. Conclusion

Adding regeneration processes into a forest gap model allowed us to quantify the relative importance of seed production and seedling survival for forest species composition. Simulating species-specific seed production and seedling survival as functions of environmental conditions improved model simulations of species composition and predictions of total basal area. In general, species in the PNW were not as sensitive to how seed production was calculated, with seedling survival being more important for species composition. Given our findings regarding the crucial role of seedling survival in shaping tree species composition, coupled with the observed insensitivity of seed production within the model, future simulations of forest dynamics should prioritize improving seedling processes and the environmental drivers that determine seedling survival.

## Data Availability

The base ForClim model can be obtained from: https://ites-fe.ethz.ch/openaccess/products/forclim. The work here updated the ForClim model and it is available upon request. Once this paper is accepted for publication it will be added to the open-access link above. Data to make the seed production equations from the MASTIF network and Wind forest dynamic plot— MASTIF network data can be obtained from: https://sites.nicholas.duke.edu/clarklab/projects [62]. WInd forest dynamic plot seed and tree data is available upon request. Weather data used for the seed production equation and parameterization of the model comes from PRISM data: https://prism.oregonstate.edu/ [63]. FIA data used for comparison and parametrization is publicly available: https://www.fia.fs.usda.gov/ [64]. Results from simulations and R code used to analyze the results are availble upon request. Once the paper is accepted for publication, the data and code will be available from the GitHub repository: https://github.com/snellOHIO [65]. Supplementary material is available online [66].
